# *Aurora*: a mobile-based cognitive behavioral therapy intervention for anxiety and depression in Mexico

**DOI:** 10.3389/fpsyg.2025.1659374

**Published:** 2025-10-10

**Authors:** Alejandro López-Tello, Adriana Pérez-Gómez, Christian Gabriel Toledo-Lozano, María del Pilar Callejas-Gómez, Raúl Durón-Figueroa, Ana Moreno-Coutiño, Antonio Ramirez-Treviño, Sebastián Nava, Diego Antonio Ocampo-Gutiérrez de Velasco, Edith Zárate, Oscar Arias-Carrión

**Affiliations:** ^1^Departamento de Psicología, Universidad Iberoamericana, Mexico City, Mexico; ^2^Private Practitioner, Mexico City, Mexico; ^3^Coordinación de Investigación, Centro Médico Nacional "20 de Noviembre", ISSSTE, Mexico City, Mexico; ^4^Laboratorio de Enseñanza Virtual y Ciberpsicología, Facultad de Psicología, Universidad Nacional Autónoma de México, Mexico City, Mexico; ^5^Laboratorio de Atención Plena Compasiva, Facultad de Psicología, Universidad Nacional de México, Mexico City, Mexico; ^6^CINVESTAV Unidad Guadalajara, Guadalajara, Mexico; ^7^Psicofarma S.A. de C.V., Mexico City, Mexico; ^8^División de Neurociencias Clínica, Instituto Nacional de Rehabilitación Luis Guillermo Ibarra Ibarra, Mexico City, Mexico; ^9^Tecnologico de Monterrey, Escuela de Medicina y Ciencias de la Salud, Mexico City, Mexico

**Keywords:** digital therapeutics, cognitive behavioral therapy, mobile health, mHealth, anxiety and depression, usability testing, low- and middle-income countries

## Abstract

**Background:**

Anxiety and depression are leading contributors to disability worldwide. In Mexico, an upper-middle-income country (UMIC) with health-system constraints resembling those of low- and middle-income countries (LMICs), access to psychotherapy remains limited. Digital therapeutics provide a scalable approach to bridging this gap. *Aurora* is a Spanish-language, self-guided mobile application delivering cognitive behavioral therapy (CBT) through eight interactive modules. This study evaluated its feasibility, usability, and preliminary clinical impact among pharmacologically treated adults with anxiety and depression.

**Methods:**

We conducted a mixed-methods, pre–post feasibility study in Mexico between March and October 2024. Participants were 46 adults (18–45 years) with psychiatrist-confirmed mild-to-moderate anxiety and/or depression on stable pharmacotherapy; 38 (82.6%) completed baseline and follow-up. Usability was assessed with the System Usability Scale (SUS) and the user Mobile App Rating Scale (uMARS). Symptoms were measured with the Spanish Goldberg Anxiety and Depression Scale (GADS). The number of modules completed defined engagement, categorized as follows: low (0–2), moderate (3–5), and high (6–7). Analyses included paired *t*-tests, non-parametric trend tests, correlations, and adjusted regressions.

**Results:**

Anxiety scores decreased from 13.2 (SD 5.6) to 10.6 (SD 5.1) (*t*(37) = 3.81, *p* = 0.001; *d* = 0.49), and depression scores from 11.1 (SD 5.2) to 8.4 (SD 4.9) (*t*(37) = 4.75, *p* < 0.001; *d* = 0.54). Engagement showed a graded dose–response: high-engagement users (*n* = 17) achieved larger reductions (Δ anxiety = −3.9; Δ depression = −4.0) than low-engagement users (Δ anxiety = −0.9; Δ depression = −1.0). Regression confirmed each additional module predicted incremental reductions in both anxiety (*β* = −0.38, *p* = 0.009) and depression (*β* = −0.41, *p* = 0.006). Usability ratings were high (SUS = 78.5/100; uMARS = 4.2/5).

**Conclusion:**

*Aurora* was feasible, usable, and associated with moderate symptom reduction in pharmacologically treated patients. The observed dose–response underscores the clinical importance of sustained engagement. These findings highlight the potential of co-designed, culturally tailored digital therapeutics to expand access to mental health care in Spanish-speaking UMIC and LMIC settings, warranting confirmation in larger controlled trials.

## 1 Introduction

Mental health disorders are a leading contributor to the global burden of disease. Anxiety and depression together account for more than 10% of all years lived with disability worldwide, affecting over 600 million people annually (GBD 2019 Diseases and Injuries Collaborators, 2020). Despite the availability of evidence-based treatments such as cognitive behavioral therapy (CBT), more than 75% of individuals in low- and middle-income countries (LMICs) receive no adequate mental health care (Patel et al., 2018).

In Mexico, anxiety and depression are the most prevalent psychiatric disorders, with lifetime prevalence estimates of 14.3 and 9.2%, respectively (Medina-Mora et al., 2005). The national health system faces significant challenges: a shortage of fewer than three psychiatrists per 100,000 inhabitants, fragmented integration between psychological and pharmacological care, stigma, and high out-of-pocket costs (Berenzon Gorn et al., 2013). The COVID-19 pandemic further intensified unmet needs by increasing psychological distress and disrupting access to in-person treatment (Hernandez-Diaz et al., 2022). Although the World Bank classifies Mexico as an upper-middle-income country (UMIC), its health system continues to share critical structural limitations with LMICs, including resource scarcity, inequitable access, and digital divides. This contextual nuance highlights that findings from Mexico can inform broader Latin American strategies, as many middle-income health systems in the region face similar constraints.

Digital therapeutics represent a promising approach to bridge this gap. Mobile health (mHealth) applications can deliver validated psychological interventions at scale, overcoming barriers of geography, stigma, and limited specialist availability (Naslund et al., 2017). Robust evidence demonstrates that digital CBT reduces anxiety and depressive symptoms across diverse populations (Firth et al., 2017; Linardon et al., 2019). In Latin America, emerging studies have confirmed the feasibility and effectiveness of smartphone-based mental health interventions (Araya et al., 2021; Karyotaki et al., 2023; Kim et al., 2023). Nevertheless, most commercially available mental health apps lack clinical validation, cultural adaptation, and regulatory oversight (Larsen et al., 2016).

*Aurora* was developed to address these shortcomings. It is a Spanish-language, self-guided mobile application delivering structured CBT through eight interactive modules. Its content—behavioral activation, cognitive restructuring, and mindfulness-based practices—was selected based on evidence linking these elements to improved outcomes in digital CBT (Cuijpers et al., 2022; Furukawa et al., 2025). Development followed a co-creation framework, incorporating clinicians, patients, and usability experts to ensure both therapeutic fidelity and user-centered design. Importantly, engagement has emerged as a consistent mediator of digital CBT outcomes, with module completion predicting greater symptom reduction (Gan et al., 2021). Assessing usability and engagement was therefore a core objective of this feasibility study.

This pilot study aimed to evaluate the feasibility, usability, and preliminary clinical impact of *Aurora* in a sample of pharmacologically treated patients with anxiety and depression in Mexico. We hypothesized that *Aurora* would be associated with reductions in self-reported symptoms, demonstrate high usability, and show a graded relationship between engagement and clinical outcomes.

## 2 Methods

### 2.1 Study design and setting

We conducted a mixed-methods, pre–post feasibility study of *Aurora*, a Spanish-language, self-guided digital therapeutic mobile application based on CBT principles. The trial was carried out in Mexico between March and October 2024 and was not powered for definitive efficacy testing. We adhered to the CONSORT extension for pilot and feasibility trials (Eldridge et al., 2016) and simulation-based recommendations for pilot sample sizes (Teare et al., 2014; Whitehead et al., 2016). A sample of 38 completers was sufficient to estimate feasibility and usability parameters, as well as symptom change variance, to inform a future randomized controlled trial (RCT). The study followed an iterative human-centered design process incorporating co-design with expert stakeholders, clinical usability testing, and pre–post evaluation of anxiety and depression symptoms.

### 2.2 Ethical considerations

The study complied with the Declaration of Helsinki and the ethical standards of the Comisión Nacional de Bioética (CONBIOÉTICA) in Mexico. The protocol (INNOVA 2024-01) received approval from the Comité de Ética en Investigación (Folio CEI-000002) and the Comité de Investigación (Folio CI-000002) of Médica Sur, S.A.B. de C.V. Review covered all procedures, including consent forms, participant interaction materials, and data protection safeguards. Written informed consent was obtained from all participants. As the intervention was a non-invasive digital therapeutic without pharmacological components, no additional physical risk was posed.

### 2.3 Expert advisory and co-creation panels

A multidisciplinary advisory board, comprising psychiatrists, psychologists, digital health specialists, and UX designers, guided the development of the app. Three activities structured this process: (i) workshops with psychiatrists and psychologists (*n* = 20) to assess clinical validity and therapeutic coherence; (ii) heuristic and accessibility evaluation by UX experts based on Nielsen’s principles, which identified issues such as excessive cognitive load, unclear navigation, and insufficient accessibility; and (iii) structured focus groups with psychiatric patients (*n* = 12) to assess emotional needs, use contexts, and perceived value. These activities informed successive redesigns of the interface and content.

### 2.4 Participants and recruitment

Participants were recruited through referrals from psychiatrists and online outreach across metropolitan areas in Mexico. Inclusion criteria were: age 18–45 years; a psychiatrist-confirmed diagnosis of mild-to-moderate anxiety and/or depression; stable pharmacotherapy for at least 1 month; and access to a smartphone with internet. Exclusion criteria were severe psychiatric comorbidity (e.g., psychosis, active suicidality) or concurrent psychotherapy. Excluding psychotherapy reduced co-intervention bias and reflected prior digital CBT trials (Johansson et al., 2019; Kambeitz-Ilankovic et al., 2022). By contrast, stable pharmacotherapy was permitted to reflect real-world practice. Of the 46 participants who initiated app use, 38 completed both pre- and post-assessments and were included in the analyses. Demographics, treatment history, and engagement were collected via digital questionnaires.

### 2.5 Intervention: *Aurora* app

*Aurora* is a Spanish-language, self-guided digital therapeutic classified as a Software as a Medical Device (SaMD) designed to complement pharmacotherapy for anxiety and depression. Its development followed human-centered design principles, informed by clinical guidelines, heuristic evaluation, and patient co-creation. The app integrates CBT elements with established digital efficacy, including cognitive restructuring, behavioral activation, and mindfulness training, which are prioritized both by the advisory board and by evidence from meta-analyses.

**Figure 1:**
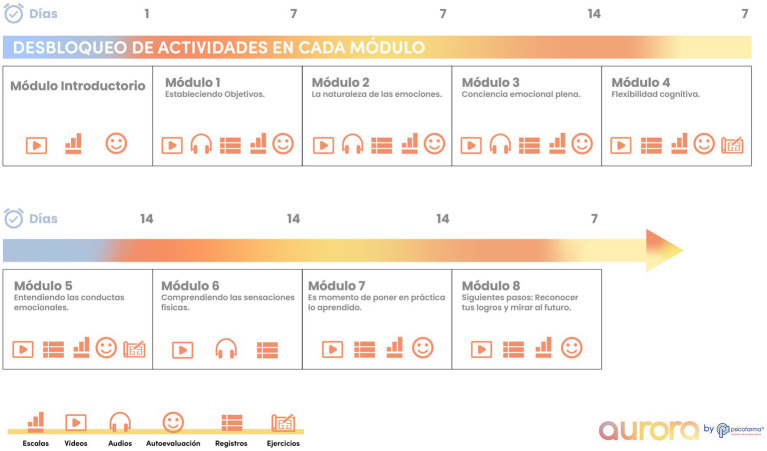
*Aurora* app architecture and therapeutic features. Schematic overview of *Aurora*, a Spanish-language, self-guided mobile application delivering cognitive behavioral therapy (CBT) for depression and anxiety. The app includes eight sequentially unlockable modules integrating breathing exercises, mindfulness training, psychoeducation, cognitive restructuring, and behavioral activation. Core functions include: (1) self-assessment tools for daily mood and symptom monitoring, (2) multimedia psychoeducational videos, (3) guided audio relaxation and CBT exercises, (4) structured activity logs and reflections, and (5) progressive unlocking of modules to encourage sustained engagement. Accessibility features include high-contrast icons, text-to-audio options, and captioned multimedia content.

**Figure 2:**
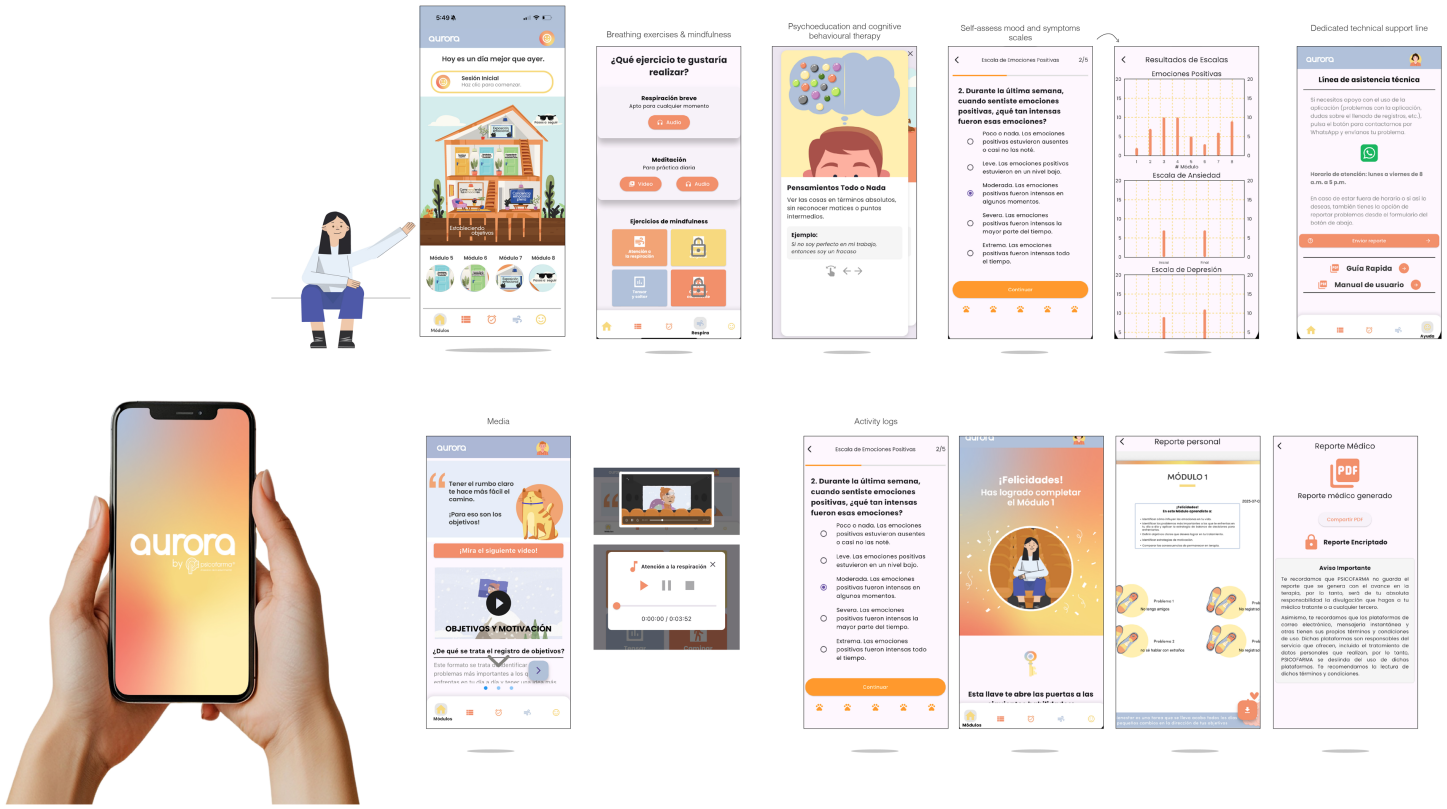
*Aurora* user interface and digital experience. Representative screenshots of the *Aurora* app, illustrating core features and user journey. Panels show: (1) the home interface with module progression, (2) breathing and mindfulness exercises, (3) psychoeducational content on cognitive restructuring, (4) self-assessment scales for anxiety, depression, and positive emotions, (5) graphical feedback of scores, (6) access to technical support, and (7) automatically generated personal and medical reports. Multimedia integration, gamified feedback, and encrypted data management support user engagement and adherence.

*Aurora* comprises eight sequentially unlockable modules targeting emotional self-regulation and cognitive restructuring (Figure 1). Modules incorporate audio-guided breathing exercises, mindfulness training, psychoeducation, behavioral activation, and structured activities to promote reframing and emotional awareness (Figure 2). At module completion, users receive personalized reports summarizing activities and symptom trajectories. Activity logs and gamified progression reinforce adherence. The interface was iteratively refined using Nielsen’s heuristics, with accessibility features such as high-contrast design, simplified icons, captions, and text-to-audio conversion. The app is compatible with both Android and iOS devices and requires continuous internet access for synchronization and to receive updated content. All data were encrypted and managed in accordance with the Mexican Federal Law on the Protection of Personal Data Held by Private Parties.

### 2.6 Outcome measures

*Primary outcomes.* Usability was assessed with the Spanish-validated System Usability Scale (SUS) (Sevilla-Gonzalez et al., 2020) and the user Mobile App Rating Scale (uMARS) (Martin-Payo et al., 2021). Qualitative feedback was thematically analyzed to identify barriers, benefits, and design improvements. Engagement was measured via in-app analytics of time, module completion, and frequency of use, and categorized into low (0–2), moderate (3–5), and high (6–7) engagement groups to test dose–response associations (Gan et al., 2021).

*Secondary outcomes.* Self-reported symptoms of anxiety and depression were measured using the Spanish Goldberg Anxiety and Depression Scale (GADS), comprising two nine-item subscales. Validated versions in Latin America were used (Espinosa et al., 2015; Martín Carbonell et al., 2016; Reivan-Ortiz et al., 2019). Internal consistency was assessed in our sample, with Cronbach’s *α* reported in the Results. Details of scale items, scoring, and validation are provided in Supplementary Table S1.

### 2.7 Statistical analysis

Analyses were conducted in Python and SPSS v27. Within-group changes in anxiety and depression were tested using paired two-tailed *t*-tests, with Cohen’s *d* and 95% CIs reported. Engagement–outcome associations were examined using Kruskal–Wallis and Jonckheere–Terpstra tests, Spearman’s *ρ*, and adjusted OLS regressions (controlling for baseline severity, age, sex). Regression models reported *β* coefficients, SEs, 95% CIs, and *p*-values. Exploratory subgroup analyses were conducted stratifying participants by age, sex, and baseline severity. Feasibility metrics included recruitment, retention, and usability scores.

## 3 Results

### 3.1 Participant characteristics

A total of 46 individuals initiated the intervention, of whom 38 (82.6%) completed both the pre- and post-assessments and were included in the final analysis. Participants were predominantly female (73.7%), aged 18–45 years (mean = 31.2 years; SD = 6.4), with the majority holding a university-level education (78.9%). All participants had been previously diagnosed with anxiety and/or depression by a psychiatrist and were undergoing pharmacological treatment during the study period (mean = 1.4 psychotropic medications; range: 1–3). No participant received concurrent psychotherapy, as this was not in line with the eligibility criteria. Baseline demographic and clinical characteristics are shown in Table 1.

**Table 1:** Baseline demographic and clinical characteristics of participants.

Characteristic	Value
Age, mean (SD), y	31.2 (6.4)
Age range, y	18–45
Female sex, *n* (%)	28 (73.7)
Education, university or higher, *n* (%)	30 (78.9)
Diagnosis, *n* (%)	Anxiety only: 14 (36.8) Depression only: 10 (26.3) Both anxiety and depression: 14 (36.8)
Duration of pharmacological treatment, mean (SD), mo	11.4 (7.8)
Psychotropic medication use, *n* (%)	38 (100)
Number of medications, mean (range)	1.4 (1–3)

The Goldberg Anxiety and Depression Scale (GADS) demonstrated acceptable internal consistency in our sample (Cronbach’s *α* = 0.82 for anxiety; 0.80 for depression). The item structure and scoring are summarized in Supplementary Table S1.

### 3.2 Usability and user experience

Usability testing with a subset of 15 users identified barriers, including excessive text density, limited interaction feedback, and inconsistencies in navigation. Iterative refinements addressed these issues.

Final usability metrics indicated high acceptability, with a mean System Usability Scale score of 78.5/100, exceeding the commonly accepted threshold for “good” usability. On the uMARS, users rated engagement, functionality, esthetics, and information quality above 4.0/5, consistent with validated benchmarks.

Participants reported that the app was easy to use, relevant to their daily lives, and aligned with their therapeutic needs.

### 3.3 Perceived impact

Thirty-one of 38 participants (81.6%) reported perceivable psychological improvements attributed to *Aurora*, particularly in emotional self-regulation, anxiety control, and integration of breathing and mindfulness exercises into daily routines. The highest perceived impact was observed among users with high engagement.

Qualitative responses reinforced quantitative findings. Representative themes included: *“learning how to manage thoughts more effectively,” “feeling calmer during the day,”* and *“feeling supported between therapy sessions.”* Common barriers included limited offline access and the desire for more personalized options.

### 3.4 Changes in anxiety and depression symptoms

Use of the *Aurora* app was associated with statistically significant and clinically meaningful reductions in self-reported symptoms of anxiety and depression (Table 2).

**Table 2:** Pre and post-changes in anxiety and depression symptoms.

Outcome	Baseline, mean (SD)	Post, mean (SD)	Mean Δ (SD)	*t*(df)	*p*-value	Cohen’s *d* (95% CI)
Anxiety score	13.2 (5.6)	10.6 (5.1)	−2.6 (3.5)	3.81 (37)	0.001	0.49 (0.22–0.76)
Depression score	11.1 (5.2)	8.4 (4.9)	−2.7 (3.4)	4.75 (37)	<0.001	0.54 (0.28–0.80)

Scores derived from the Spanish Goldberg Anxiety and Depression Scale. Higher scores reflect greater symptom severity.

**Figure 3:**
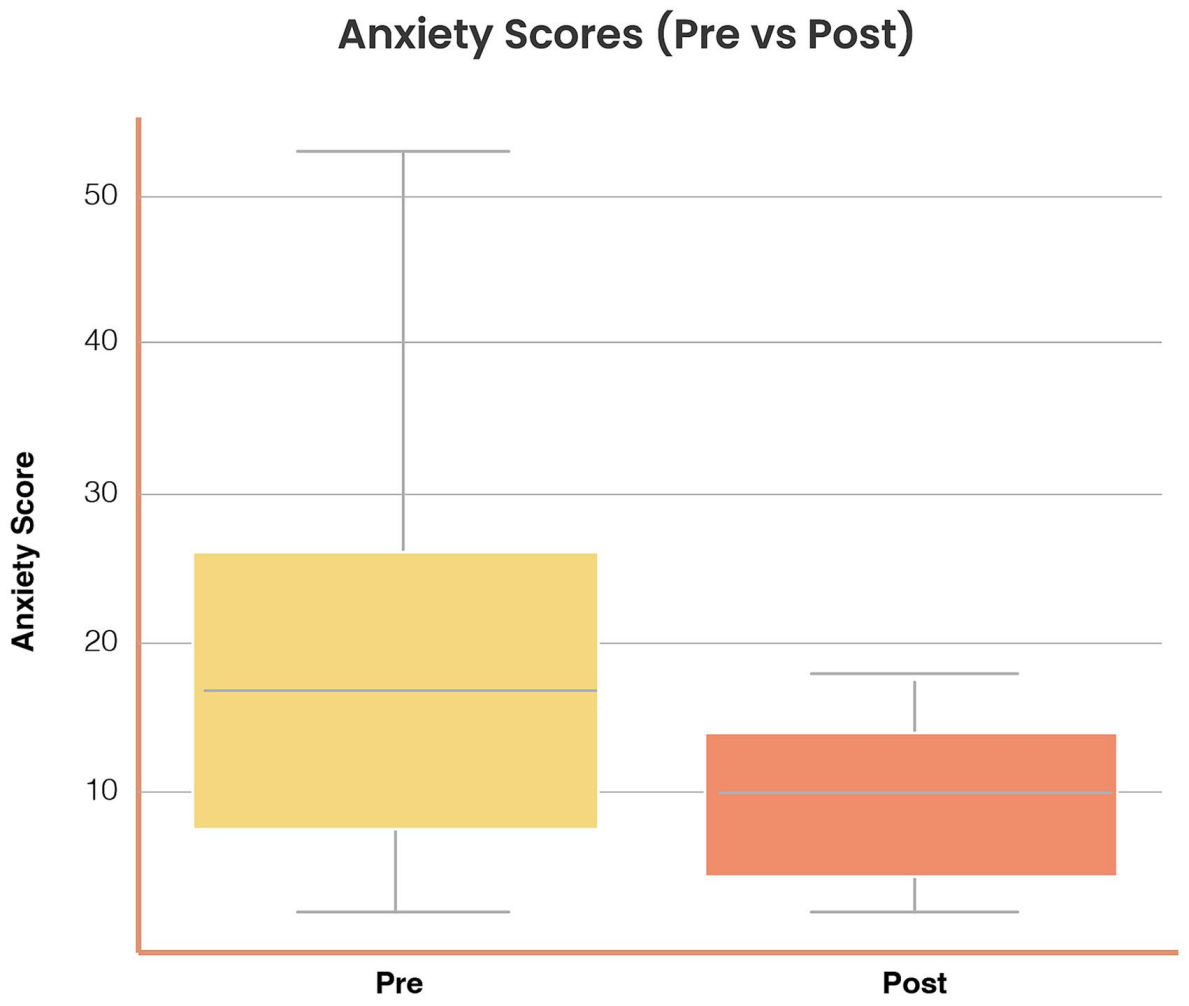
Reductions in anxiety symptoms following *Aurora* use. Distribution of anxiety scores (Goldberg Anxiety subscale, 0–21) at baseline (Pre) and post-intervention (Post). Mean anxiety scores decreased from 13.2 (SD = 5.6) to 10.6 (SD = 5.1), Δ = −2.6 (SD = 3.5), *t*(37) = 3.81, *p* = 0.001, Cohen’s *d* = 0.49 (95% CI [0.22, 0.76]). Boxplots display medians, interquartile ranges, and outliers. Symptom reductions were consistent across age and gender strata.

*Anxiety symptoms:* Mean scores decreased from 13.2 (SD = 5.6) at baseline to 10.6 (SD = 5.1) post-intervention (Δ = −2.6, SD = 3.5), *t*(37) = 3.81, *p* = 0.001, Cohen’s *d* = 0.49, 95% CI [0.22, 0.76]. Figure 3 illustrates pre–post distributions.

**Figure 4:**
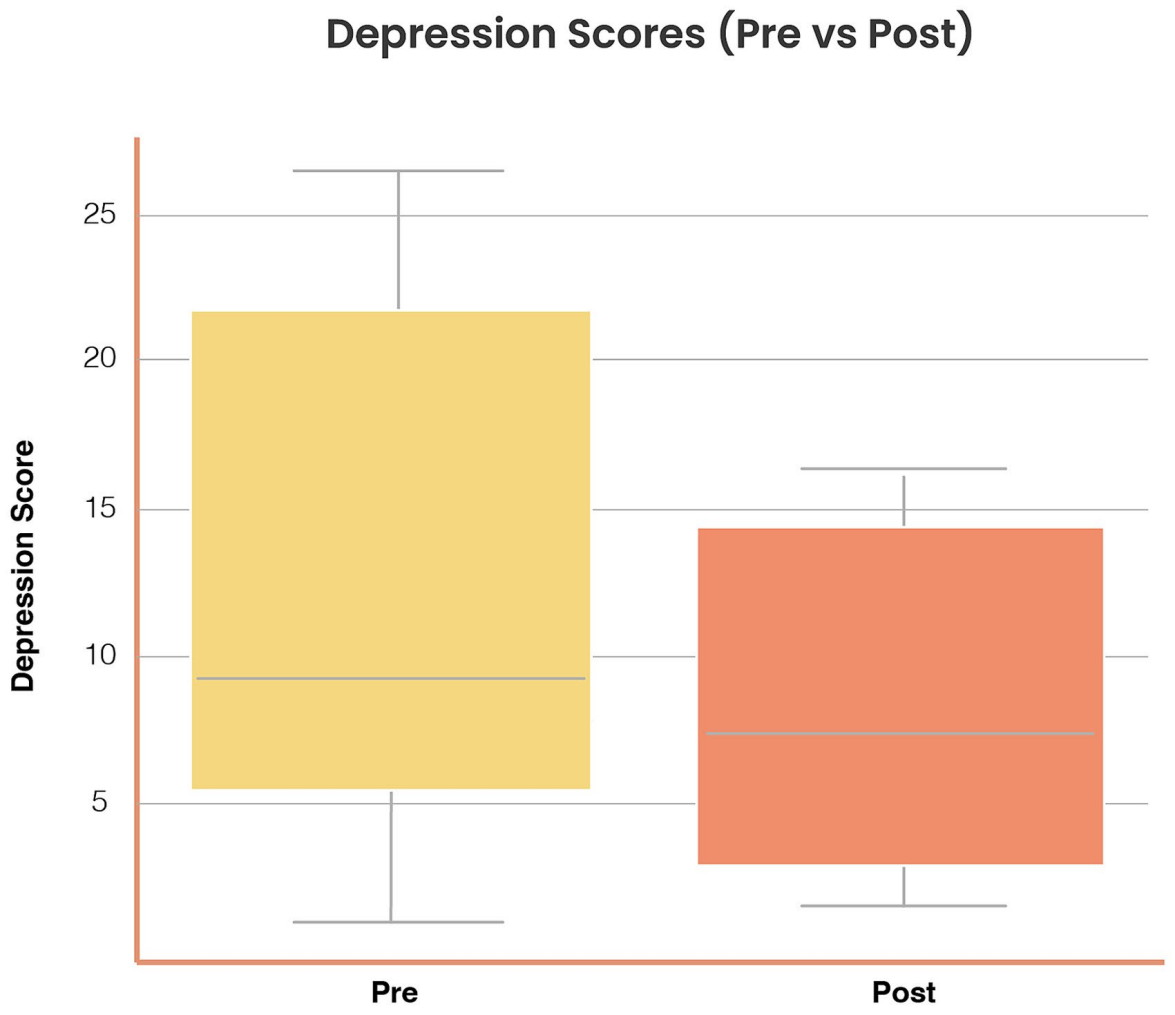
Reductions in depression symptoms following *Aurora* use. Distribution of depression scores (Goldberg Depression subscale, 0–21) at baseline and post-intervention. Mean depression scores decreased from 11.1 (SD = 5.2) to 8.4 (SD = 4.9), Δ = −2.7 (SD = 3.4), *t*(37) = 4.75, *p* < 0.001, Cohen’s *d* = 0.54 (95% CI [0.28, 0.80]). Boxplots show distributions with consistent reductions across subgroups.

*Depression symptoms:* Mean scores decreased from 11.1 (SD = 5.2) to 8.4 (SD = 4.9) (Δ = −2.7, SD = 3.4), *t*(37) = 4.75, *p* < 0.001, Cohen’s *d* = 0.54, 95% CI [0.28, 0.80]. Figure 4 illustrates pre–post distributions.

Symptom improvements were consistent across subgroups stratified by age and gender, suggesting the broad applicability of *Aurora* across various user profiles.

### 3.5 App engagement profiles

Based on the number of modules completed, participants were categorized into three engagement groups: low (0–2 modules, *n* = 9), moderate (3–5 modules, *n* = 12), and high engagement (6–7 modules, *n* = 17). The median total time dedicated to in-app activities was 98 min (range, 22–193 min).

*Dose–response analysis* demonstrated a graded association between engagement and clinical benefit. Compared with the low-engagement group, participants with high engagement reported significantly larger reductions in both anxiety (Kruskal–Wallis *H* = 7.82, *p* = 0.02) and depression (*H* = 9.14, *p* = 0.01). A Jonckheere–Terpstra test confirmed an ordered trend (*p* = 0.01 for anxiety; *p* = 0.008 for depression).

**Figure 5:**
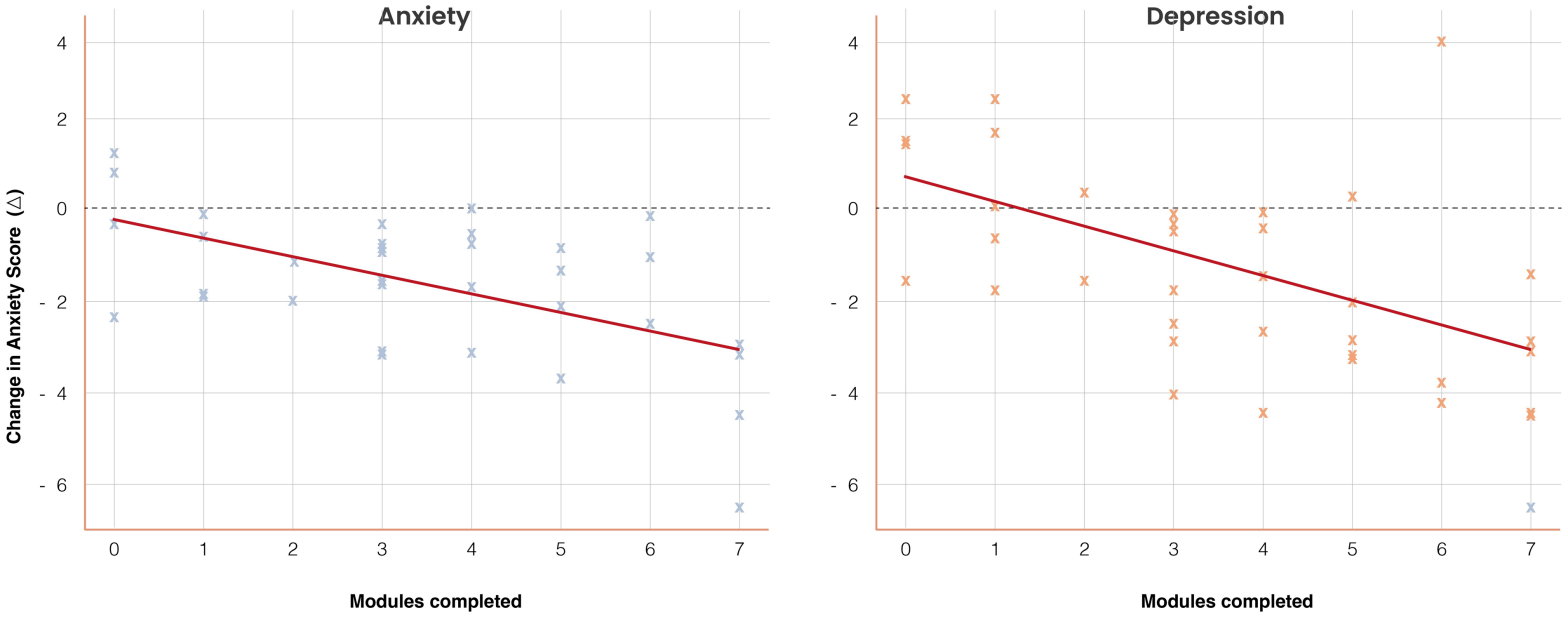
Dose–response association between engagement and symptom change. Scatterplots with regression lines illustrate the relationship between the number of modules completed (*x*-axis) and changes in anxiety and depression scores (*y*-axis). Higher engagement predicted greater symptom reduction: anxiety *β* = −0.38 (SE = 0.14), *p* = 0.009; depression *β* = −0.41 (SE = 0.15), *p* = 0.006. Shaded regions represent 95% confidence intervals. Group means (low = 0–2, moderate = 3–5, high = 6–7 modules) are overlaid for comparison. Results demonstrate a graded dose–response effect.

Spearman’s correlations indicated significant associations between modules completed and change scores (*ρ* = −0.41, *p* = 0.01 for anxiety; *ρ* = −0.44, *p* = 0.007 for depression). In adjusted OLS regression, each additional completed module predicted a mean reduction of 0.38 points in anxiety (*β* = −0.38, SE = 0.14, 95% CI [−0.66, −0.10], *p* = 0.009) and 0.41 points in depression (*β* = −0.41, SE = 0.15, 95% CI [−0.71, −0.12], *p* = 0.006). These results are detailed in Supplementary Tables S2–S3 and illustrated in Figure 5.

## 4 Discussion

This feasibility study provides preliminary evidence that *Aurora*, a culturally adapted, Spanish-language mobile application delivering CBT, is usable, acceptable, and clinically relevant for patients with anxiety and depression in Mexico. Among participants on stable pharmacotherapy, the app was associated with moderate reductions in self-reported symptoms, high usability ratings, and a precise dose–response between engagement and outcomes. These findings extend the literature on digital therapeutics, which has been predominantly shaped by evidence from high-income countries, to an underrepresented Latin American context.

A central strength of this work is the co-creation methodology, which integrated clinicians, patients, and usability experts from the outset. This process ensured therapeutic fidelity while addressing common shortcomings of commercial mental health apps, including excessive cognitive load and inadequate cultural adaptation. The observed usability scores (mean SUS = 78.5/100; uMARS = 4.2/5) compare favorably with benchmarks reported in digital CBT interventions from high-income countries (Firth et al., 2017; Linardon et al., 2019), underscoring the potential for deployment in Spanish-speaking health systems with resource limitations.

The dose–response association between module completion and symptom reduction strengthens the case for *Aurora’s* clinical relevance. Participants in the high-engagement group achieved reductions of nearly four points on both anxiety and depression subscales, compared with minimal change in the low-engagement group. Regression analyses confirmed that each additional module completed predicted incremental symptom improvement, independent of baseline severity, age, and sex. This finding is consistent with meta-analytic results indicating that the intensity of use is a key mediator of digital CBT outcomes (Furukawa et al., 2025; Gan et al., 2021). Engagement thus emerges as a modifiable determinant of clinical benefit, with implications for future app design and implementation strategies.

This study also contributes to the debate on the external validity of digital therapeutics in UMICs. Although Mexico is classified as a UMIC, its mental health system continues to share critical constraints with LMICs, including workforce shortages, stigma, fragmented care, and high out-of-pocket costs. By situating our findings within this broader context, we show how scalable digital solutions may help bridge structural gaps across Latin America. Evidence from pragmatic trials in Chile, Brazil, and other LMICs has similarly demonstrated the feasibility and acceptability of mobile CBT interventions in under-resourced environments (Araya et al., 2021; Karyotaki et al., 2023; Kim et al., 2023).

Several limitations should temper interpretation. First, the absence of a control group precludes causal inference; symptom reductions may partly reflect regression to the mean, expectancy effects, or concurrent pharmacotherapy. Second, although all participants were receiving medication (mean, 1.4 drugs; range, 1–3), outcomes were not stratified by pharmacological class. Exploratory sensitivity analyses suggest that engagement–outcome associations were robust to medication count, but larger studies are needed to evaluate interactions between pharmacotherapy and digital CBT. Third, data on illness chronicity were not collected. Chronicity may moderate treatment response, and future trials should include this variable (Cuijpers et al., 2022; Kambeitz-Ilankovic et al., 2022; Haller et al., 2023; Sextl-Plotz et al., 2024). Fourth, although the Spanish GADS has been validated in Mexico and other Latin American populations (Espinosa et al., 2015; Martín Carbonell et al., 2016; Reivan-Ortiz et al., 2019), the minor linguistic adaptations applied here were not formally re-validated. We confirmed good internal consistency in this sample; however, psychometric validation in Mexican populations remains necessary. Finally, participants were predominantly urban and highly educated, which may limit the generalizability of the findings to rural or underserved populations.

Despite these limitations, the findings carry important implications. First, the results demonstrate that a self-guided CBT app can deliver clinically meaningful benefits as an adjunct to pharmacotherapy, supporting its potential role in integrated care. Second, the graded dose–response highlights the importance of engagement, suggesting that strategies such as gamification, reminders, or integration with telepsychiatry platforms could enhance effectiveness. Third, the co-creation framework—balancing clinical fidelity with user-centered design—offers a scalable model for adapting digital therapeutics to diverse cultural contexts.

Future research should prioritize adequately powered, randomized controlled trials with active comparators, longer follow-up periods, and clinician-rated outcomes. A collection of chronicity, medication regimens, and sociodemographic moderators will help identify who benefits most. Broader linguistic and cultural adaptation will also be essential before *Aurora* can be scaled across Latin America, but its modular architecture facilitates such expansion.

In conclusion, *Aurora* represents a feasible, usable, and culturally tailored digital therapeutic for anxiety and depression in Mexico. By combining evidence-based CBT with rigorous co-design and usability testing, it offers a scalable strategy to reduce treatment gaps in Spanish-speaking middle-income settings. If validated in larger controlled trials, *Aurora* and similar interventions could contribute to addressing one of the most urgent challenges in global mental health: the persistent inequity in access to effective treatment for common psychiatric disorders.

## Data Availability

The raw data supporting the conclusions of this article will be made available by the authors, without undue reservation.
