# Supplementary material for: Aurora: a mobile-based cognitive behavioral therapy intervention for anxiety and depression in Mexico
Source: Front Psychol. 2025 Oct 10;16:1659374. doi: 10.3389/fpsyg.2025.1659374 (PMC12550774; doi:10.3389/fpsyg.2025.1659374)
Supplement: Supplementary file 1 [file Table_1.docx]

## *****Supplementary Tables*****

### *****Supplementary Table S1*.** Structure and scoring of the Spanish Goldberg Anxiety and Depression Scale (GADS).**

| Domain | Items (binary: Yes/No) | Score range | Scoring notes |
| --- | --- | --- | --- |
| Anxiety | 9 items (screening + probe) | 0–9 | Score ≥4 suggests clinically significant anxiety symptoms |
| Depression | 9 items (screening + probe) | 0–9 | Score ≥2 suggests clinically significant depressive symptoms |
| Severity extension | Converted to Likert-type in *Aurora* app (0–3 scale per item) | 0–21 | Higher scores = greater severity |
| Validation | Spanish versions validated in Mexico. | — | Cronbach’s α in the present sample: Anxiety = 0.82; Depression = 0.80 |

### *****Supplementary Table S2.*** Symptom change stratified by engagement group**

| Engagement group | n | Anxiety Δ mean (SD) | Depression Δ mean (SD) |
| --- | --- | --- | --- |
| Low (0–2 modules) | 9 | –0.9 (2.1) | –1.0 (2.3) |
| Moderate (3–5) | 12 | –2.4 (3.2) | –2.5 (2.9) |
| High (6–7) | 17 | –3.9 (3.8) | –4.0 (3.2) |
| ****Trend test (p)**** | — | 0.01 | 0.008 |

Note: Jonckheere–Terpstra test confirmed ordered dose–response association across groups.

### *****Supplementary Table S3.*** Regression analysis of engagement–outcome associations**

| Predictor | Outcome | β (SE) | 95% CI | p-value |
| --- | --- | --- | --- | --- |
| Modules completed | Anxiety Δ | –0.38 (0.14) | –0.66 to –0.10 | 0.009 |
| Modules completed | Depression Δ | –0.41 (0.15) | –0.71 to –0.12 | 0.006 |
| Age (y) | Anxiety Δ | –0.05 (0.04) | –0.13 to 0.03 | 0.22 |
| Female sex (vs male) | Depression Δ | –0.12 (0.37) | –0.86 to 0.61 | 0.73 |
| Baseline severity (per 1 SD) | Anxiety Δ | –0.24 (0.12) | –0.49 to 0.01 | 0.06 |

Note: OLS regression adjusted for baseline severity, age, and sex. A negative β indicates a greater reduction in symptom scores with a higher predictor value.
